# An integrated computational approach towards novel drugs discovery against polyketide synthase 13 thioesterase domain of *Mycobacterium tuberculosis*

**DOI:** 10.1038/s41598-023-34222-8

**Published:** 2023-04-28

**Authors:** Ali Altharawi, Manal A. Alossaimi, Mohammed M. Alanazi, Safar M. Alqahatani, Muhammad Tahir ul Qamar

**Affiliations:** 1 Department of Pharmaceutical Chemistry, College of Pharmacy, Prince Sattam Bin Abdulaziz University, Al-Kharj, 11942 Saudi Arabia; 2 Department of Pharmaceutical Chemistry, College of Pharmacy, King Saud University, Riyadh, 11451 Saudi Arabia; 3 Department of Bioinformatics and Biotechnology, Government College University Faisalabad (GCUF), Faisalabad, 38000 Pakistan

**Keywords:** Virtual drug screening, Tuberculosis

## Abstract

The acquired drug resistance by *Mycobacterium tuberculosis (M. tuberculosis)* to antibiotics urges the need for developing novel anti-*M. tuberculosis* drugs that possess novel mechanism of action. Since traditional drug discovery is a labor-intensive and costly process, computer aided drug design is highly appreciated tool as it speeds up and lower the cost of drug development process. Herein, Asinex antibacterial compounds were virtually screened against thioesterase domain of Polyketide synthase 13, a unique enzyme that forms α-alkyl β-ketoesters as a direct precursor of mycolic acids which are essential components of the lipid-rich cell wall of *M. tuberculosis*. The study identified three drug-like compounds as the most promising leads; BBB_26582140, BBD_30878599 and BBC_29956160 with binding energy value of − 11.25 kcal/mol, − 9.87 kcal/mol and − 9.33 kcal/mol, respectively. The control molecule binding energy score is -9.25 kcal/mol. Also, the docked complexes were dynamically stable with maximum root mean square deviation (RMSD) value of 3 Å. Similarly, the MM-GB\PBSA method revealed highly stable complexes with mean energy values < − 75 kcal/mol for all three systems. The net binding energy scores are validated by WaterSwap and entropy energy analysis. Furthermore, The in silico druglike and pharmacokinetic investigation revealed that the compounds could be suitable candidates for additional experimentations. In summary, the study findings are significant, and the compounds may be used in experimental validation pipeline to develop potential drugs against drug-resistant tuberculosis.

## Introduction

Tuberculosis (TB) is a deadly infectious disease caused by *Mycobacterium tuberculosis (M. tuberculosis)* and listed as the 13th leading cause of mortality worldwide and the 2nd infectious leading killer after COVID-19. According to the WHO, it was estimated that 10 million people were infected with TB in 2020, of which 1.5 million people died. Global reports indicate that the incidence rate is rising in all countries and among all ages and further aggravated by latent infections. Furthermore, it is estimated that 5–10% of world population will develop active TB during their lifetime^1^. The standard treatment for drug sensitive TB requires 6–9 months regimen of antibiotics combination. Essentially, four first-line antibiotics used in the treatment of TB (isoniazid–INH, rifampicin–RMP, ethambutol–EMB, pyrazinamide–PZA) and some other drugs which can be administered to patients infected with resistant strains *M. tuberculosis* Although TB is curable and preventable, multidrug-resistant TB (MDR-TB) and extensively drug-resistant strains of *M. tuberculosis* (XDR-TB) remain a global burden rendering the standard treatment ineffective^2^. It is estimated that about 0.5 million of MDR-TB cases reported annually worldwide and nearly 14,000 XDR-TB cases were originated from 81 countries^3,4^. Additionally, the management of TB was further aggravated as MDR-TB developed resistant to two of the first-line drugs; RMP and INH, while XDR-TB is resistant to the four essential antitubercular drugs and at least one of the injectable second-line drugs such as amikacin, capreomycin, or kanamycin^5,6^. The current guideline of treating MDR-TB and XDR-TB involve pretomanid, and bedaquiline (approved by the US FDA in 2019 and 2012, respectively) along with linezolid as the first line tuberculosis therapy^1,7^. In consequence to the high number of TB resistant cases, the demand to introduce promising antitubercular drugs are urgently needed particularly those that are designed to target tuberculosis biomolecules essential for various biological processes.

The cell wall of *M. tuberculosis* is regarded a rich source of molecular targets that can be exploited to design and develop novel antitubercular drugs^8,9^. It is a unique structure that contains mycomembrane and peptidoglycan layer. The former comprised long chains of fatty acids termed as mycolic acids (MAs). These molecules are specific lipid components that are essential for the survival and virulence of *M. tuberculosis* and bind to arabinogalactan via ester bonds^10,11^. The inhibition of MAs biosynthesis is the main mechanism for the effect of the forefront and effective anti-tubercular drug INH^12^. Hence, much interest have been devoted to deciphering the biosynthesis of MAs to recruit attractive targets for development of unconventional antitubercular drugs to tackle the drug-resistant TB.

Polyketide synthase 13 (Pks13) plays a crucial role in the biosynthesis of MAs in which it mediates the condensation of two fatty acids to form mycolic β-ketoester, a direct precursor for MAs. It comprises five distinct catalytic domains, of which thioesterase domain (TE) plays a dual function where it first acts as a hydrolase to break the thioester bond and form an ester bond between mycolic β-ketoesters and the hydroxyl group of Ser1533 of the TE domain. In the second function, TE acts as an acyltransferase to form trehalose monomycolate (TMM) which is then transported and attached to arabinogalactan^13,14^. The thioesterase domain of Pks13 (Pks13-TE) has been recognized as a druggable target for developing anti-tubercular drugs^15,16^. As a result, several potential compounds have been proposed to interact with the Pks13-TE. These compounds include thiophenes^17^, benzofurans^18^, and β-lactones^19^.

Conventional drug development is a daunting, time-consuming, and costly task that suffers from high failure rate^20–23^. In recent times, the computer aided drug design (CADD) techniques have gained considerable attention and proved indispensable in drug discovery process^24^. CADD methods are in silico approach that can accelerate the drug discovery and shorten the time needed for leads identification to drug marketing. Additionally, these computational methods are critical in estimating biological activities of chemical compounds against any given biological target^25,26^. CADD can also be helpful in determining the binding affinity of compounds when it is docked to any target of interest as well as predicting the physicochemical properties of compounds^27–31^. The CAAD methods have successfully used in the past for discovery of drugs such as nelfinavir, imatinib, and zanamivir^32^. Considering the vast applications of CADD techniques in drug discovery, we herein targeted Pks13-TE domain using a multi-pronged in silico approach. The findings gathered in this study might be useful in successful identification of leads that can be further optimized for biological activity as novel anti-tubercular therapeutics.

## Materials and methods

The study was based on in silico techniques that can be split into following phases;

### Asinex library preparation

The Asinex antibacterial library was retrieved from Asinex web available at https://www.asinex.com/. The library contains 6208 compounds of natural product-like scaffolds providing diversity and accessibility for experimental studies. The library was imported to LigandScout software^33^ where the library was filtered based on Lipinski rule of five^34^. The filtered library was then subjected to PyRx 0.8^35^ to energy minimized the compounds using MM2 force field^36^ followed by conversion to .pdbqt format to make the compounds ready for docking studies.

### Pks13-TE enzyme preparation

For potential leads identification against Pks13-TE, a protein data bank (PDB) ID of “7M7V” was chosen based on the fact that the structure is most recent with good resolution of 2.29 Å^37^. The enzyme structure was then subjected to energy minimization process in UCSF Chimera 1.16^38^. The energy minimization process was done in two steps: first by steepest descent for 1000 steps followed by conjugate gradient algorithm for 1500 rounds. Missing hydrogen atoms were added to the enzyme and charge assignment was done through gasteiger method. The RMSD of minimized and non-minimized Pks13-TE was 0.24 Å. After energy minimization, the enzyme was saved into .pdb format and converted afterward into .pdbqt format for utilization in virtual docking studies.

### Structure based virtual screening process

To identify potential leads against Pks13-TE, structure based virtual screening process was performed. This was done using coordinates information of Tyr1663 which is considered a focus point of the thioesterase domain. The virtual screening process was achieved using PyRx 0.8 by employing AutoDock Vina 4.0^35,39^. The grid box was centered covering coordinates of 4.32 Å (x-axis), 35.85 Å (y-axis) and 6.77 Å (z-axis) with dimensions of 25 Å on XYZ planes. Each molecule in the library was docked with the enzyme 100 times and the best posed molecules were ranked based on binding energy score which was measured in kcal/mol. Molecules with lowest binding energy score in reference to the control molecule, Benzofuran 1^37^, were reported. The control molecules extracted from the crystal structure and redocked to the enzyme to validate the docking procedure. Another round of docking procedure revalidation was achieved by using GOLD docking software considering the same set of parameters described above. The best docked complexes were used in Discovery Studio Visualizer 2021 to examine the docked intermolecular conformation and observed chemical interactions network^40^.

### Molecular dynamic simulation (MDS)

MDS was done to get information about physical movements of docked complexes. The MDS was performed using AMBER20 package^41^. The complexes were processed through Antechamber program^42^. For compounds, Amber general force field (GAFF) was employed to generate parameter files while for Pks13-TE, FF14SB was considered^43,44^. The systems were solvated into TIP3P water model and then counter ions were added to neutralize the net charge. The padding distance set between the complex and water box edge was 12 Å^45–47^. Energy minimization was then carried out for complexes using steepest descent and conjugate gradient algorithms for total of 3000 steps. After that, the complexes were heated to 310 K for 500 ps. The systems were then equilibrated and subjected to production run of 300 ns. This time scale was found sufficient to get well converged systems. The long term interactions were treated via particle mesh Ewald method^48^. The hydrogen bonds were constrained via SHAKE algorithm while for temperature control, Langevin algorithm was used^49,50^. The simulation trajectories processed through CPPTRAJ module to investigate structure stability of the systems^51^. Plots were made using XMGRACE^52^. Further, MM-GB\PSA binding free energy was employed to get confidence on the intermolecular binding affinity^53,54^. For this purpose, 1500 frames were opted from the entire simulation trajectories at equal time interval to investigate intermolecular binding energies of complexes along the simulation time length. The extracted frames were subjected to MMPBSA.py script to estimate net binding free energy^55^. The net binding free energy was estimated through the following equation;

$$ \Delta G_{{{\text{binding}}}} = \, \Delta G_{{{\text{complex}}}} {-} \, G_{{{\text{protein}}}} - \, G_{{{\text{ligand}}}} $$

Validation on the MM-GB\PSA was done through more sophisticated WaterSwap method that uses reaction coordinates to swap water cluster equal to size of the ligand molecule and swap it with the ligand^56^. The WaterSwap was defaulted for 1000 iterations. Three algorithms were used in WaterSwap: thermodynamic integration, Bennett’s, and free energy perturbation.

### Entropy energy analysis

The binding entropy energy of docked compound was also estimated to get real binding affinity score of complexes. For this, AMBER normal mode analysis was employed^54^. The method is computationally expensive; therefore, only several snapshots (10 frames) from the entire simulation trajectories were selected. In this method, several energies were determined such as translational, vibrational and rotational^57,58^.

### SwissADME and pkCSM analysis

The computational druglikeness and pharmacokinetics were predicted to ensure selection of only promising molecules^59,60^. The absorption, distribution, metabolism, and excretion (ADME) and toxicity of compounds were estimated using SwissADME and pkCSM^28,61^.

## Results and discussion

### Docking studies

**Figure 1:**
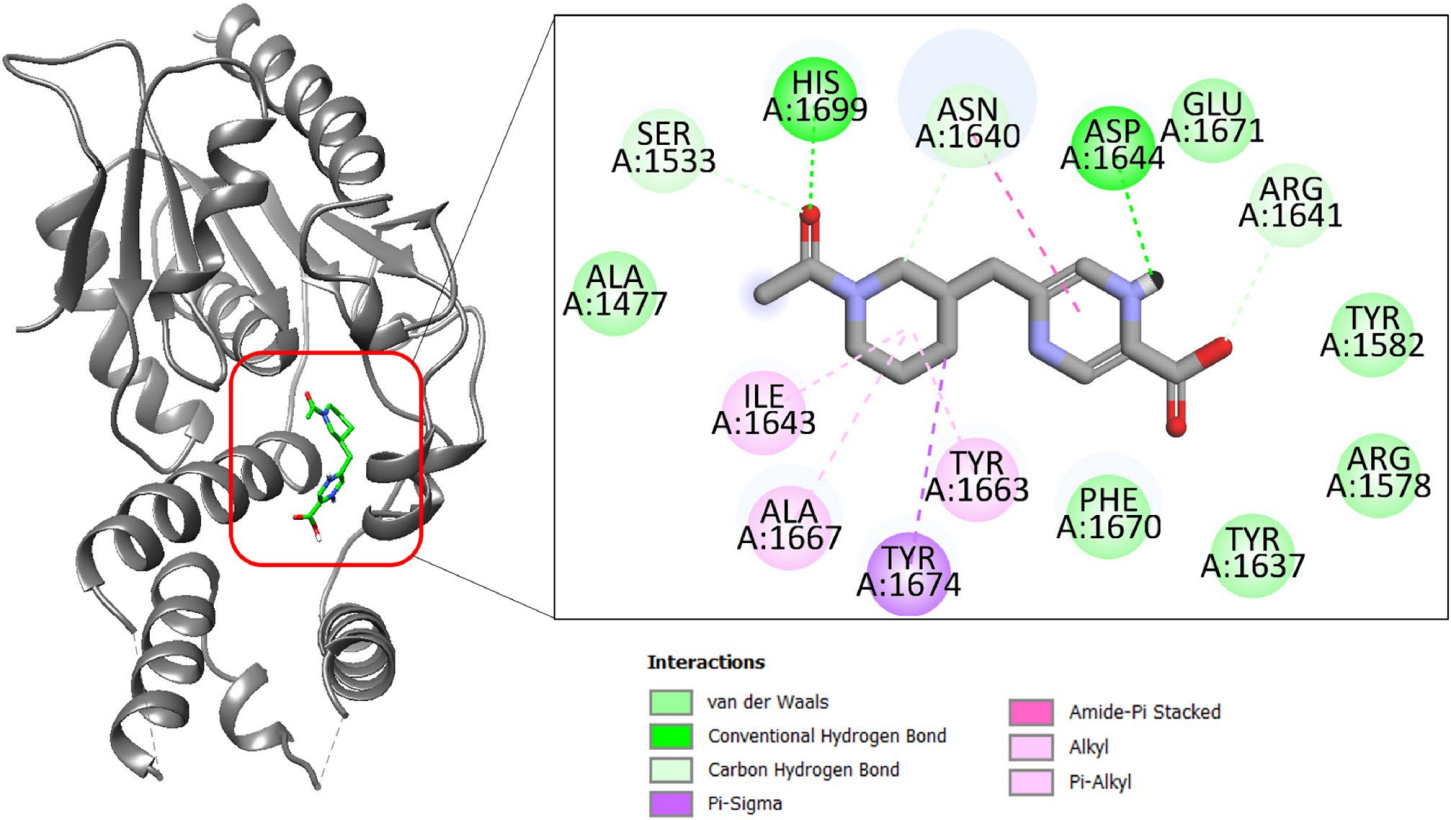
Binding mode of compound BBB_26582140 at the active pocket of polyketide synthase Pks13. The receptor enzyme is given in gray cartoon while the compound is presented in green stick. The binding interactions network is also provided.

Structure based virtual screening was conducted to prioritized chemical compound that bind best to the Pks13-TE. The combined approach of virtual screening and MDS have been used in the past to successfully screen inhibitor molecules against biological targets^45,62–64^. The Lipinski rule of five revealed total of 26,024 molecules as druglike compounds. These molecules were used in virtual screening process against Pks13-TE. Three compounds have stable binding affinity and identified as promising leads. These compounds are BBB_26582140, BBD_30878599 and BBC_29956160 with binding energy value of − 11.25 kcal/mol, − 9.87 kcal/mol and − 9.33 kcal/mol, respectively. The control molecule binding energy score was − 9.25 kcal/mol. The binding affinity of the compounds/control at the active pocket of polyketide synthase Pks13 was revisited by GOLD docking software. The GOLD fitness score of BBB_26582140 was 78.62, BBD_30878599 has GOLD score of 75.83 while the GOLD docked affinity of BBC_29956160 was 72.55. The control molecule secures a GOLD fitness score of 73.00. The BBB_26582140 is 2-((1-acetylpiperidin-3-yl)methyl)-5-carboxypyrazine-1,4-diium. The compounds achieved deep floor binding at Pks13 functional domain and accomplished rich interacting network. The oxygen atom of 1-(piperidin-1-yl)ethanone formed closed distance hydrogen bond of 2.01 Å with His1699. Another hydrogen bond was revealed with Asp1644 at distance of 1.88 Å via 2-carboxypyrazine-1,4-diium ring. The binding mode and interactions of compound BBB_26582140 is given in Fig. 1.

**Figure 2:**
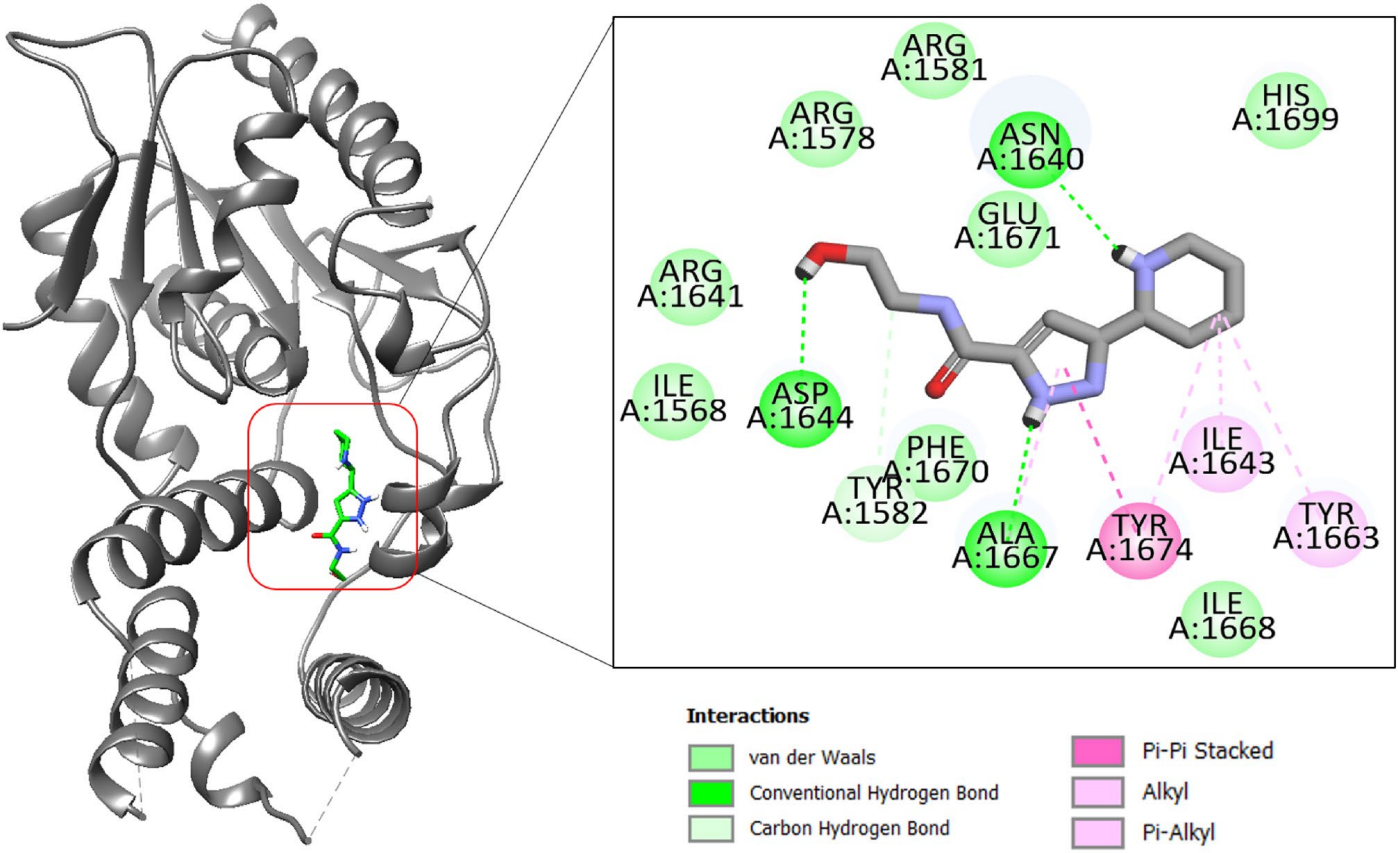
Binding mode of compound BBD_30878599 at the active pocket of polyketide synthase Pks13. The receptor enzyme is given in gray cartoon while the compound is presented in green stick. The binding interactions network is also provided.

The BBD_30878599 is N-(2-hydroxyethyl)-5-(piperidin-2-yl)-2,5-dihydro-1H-pyrazole-3-carboxamide. The compound interactions are balanced by both van der Waals and hydrogen bonds. The N-(2-hydroxyethyl)formamide is mainly engaged in hydrogen bonds with Asp1644 and Ala1667. The compounds like the first compound docked deep inside the pocket and gained access to the pocket bottom (Fig. 2).

**Figure 3:**
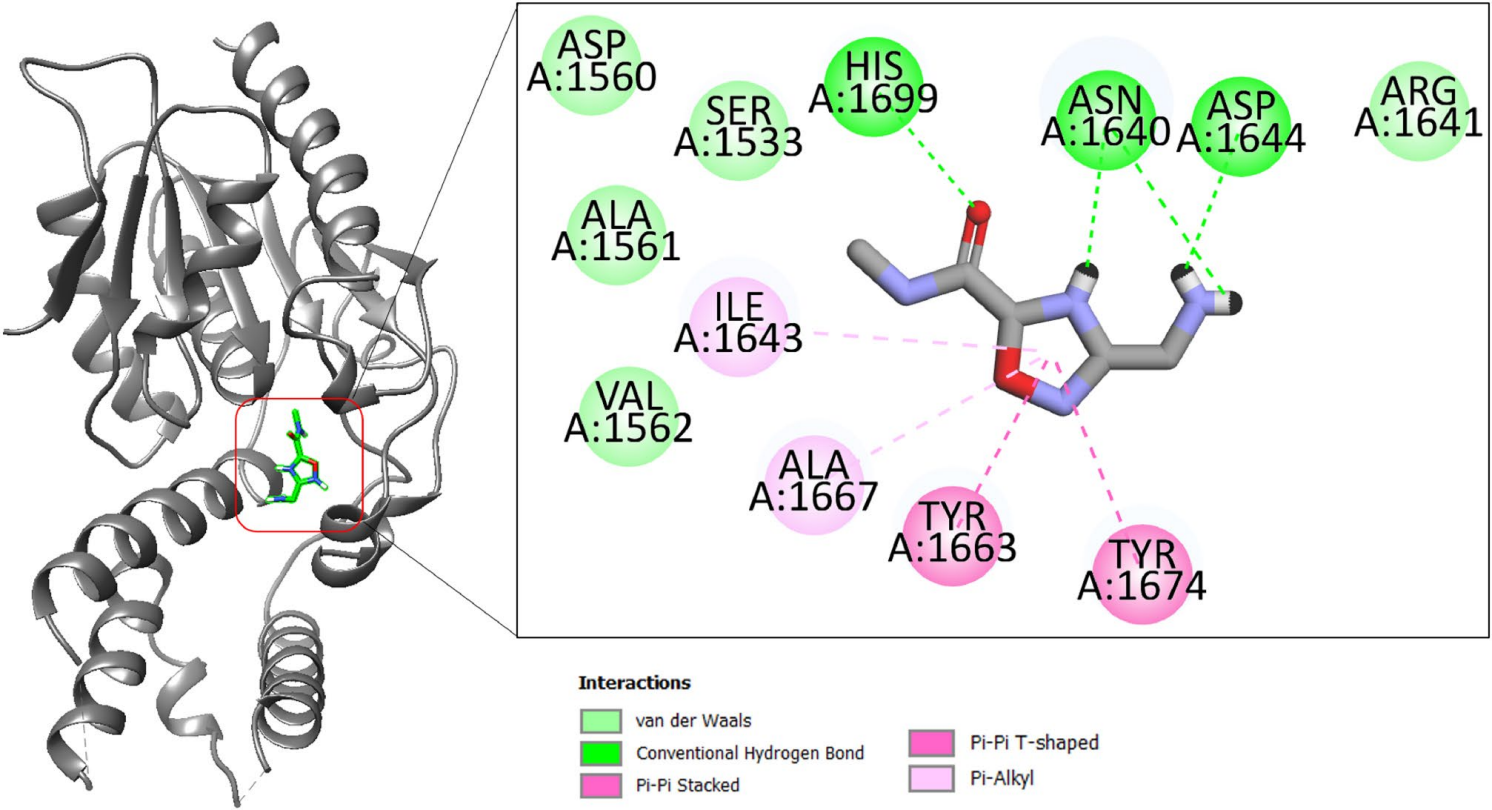
Binding mode of compound BBC_29956160 at the active pocket of polyketide synthase Pks13. The receptor enzyme is given in gray cartoon while the compound is presented in green stick. The binding interactions network is also provided.

The BBC_29956160 is 3-(aminomethyl)-N-methyl-2,5-dihydro-1,2,4-oxadiazole-5-carboxamide. This compound accommodates across the pocket length and forms hydrogen bond with Asp1644 at distance of 1.87 Å. Also, the compound produces His1699 and Asn1640 at bond distance of 2.63 Å and 1.91 Å, respectively (Fig. 3).

**Figure 4:**
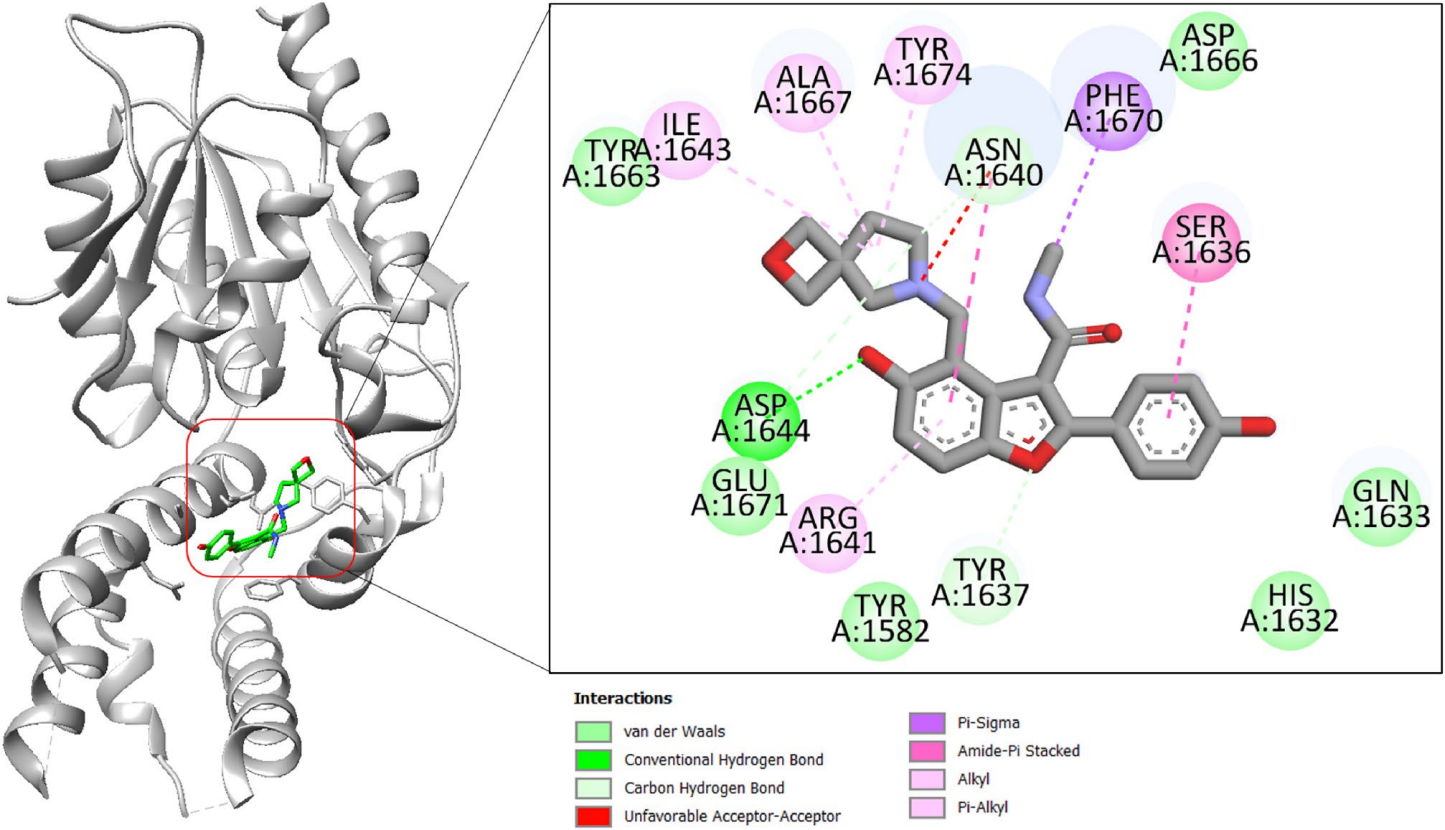
Binding mode of control compound at the active pocket of polyketide synthase Pks13. The receptor enzyme is given in gray cartoon while the compound is presented in green stick. The binding interactions network is also provided.

The control compound interactions with the enzyme is mainly dominated by hydrophobic contact and only one hydrogen bond was witnessed with Asp1644 (Fig. 4).

### Dynamic studies of docked complexes

**Figure 5:**
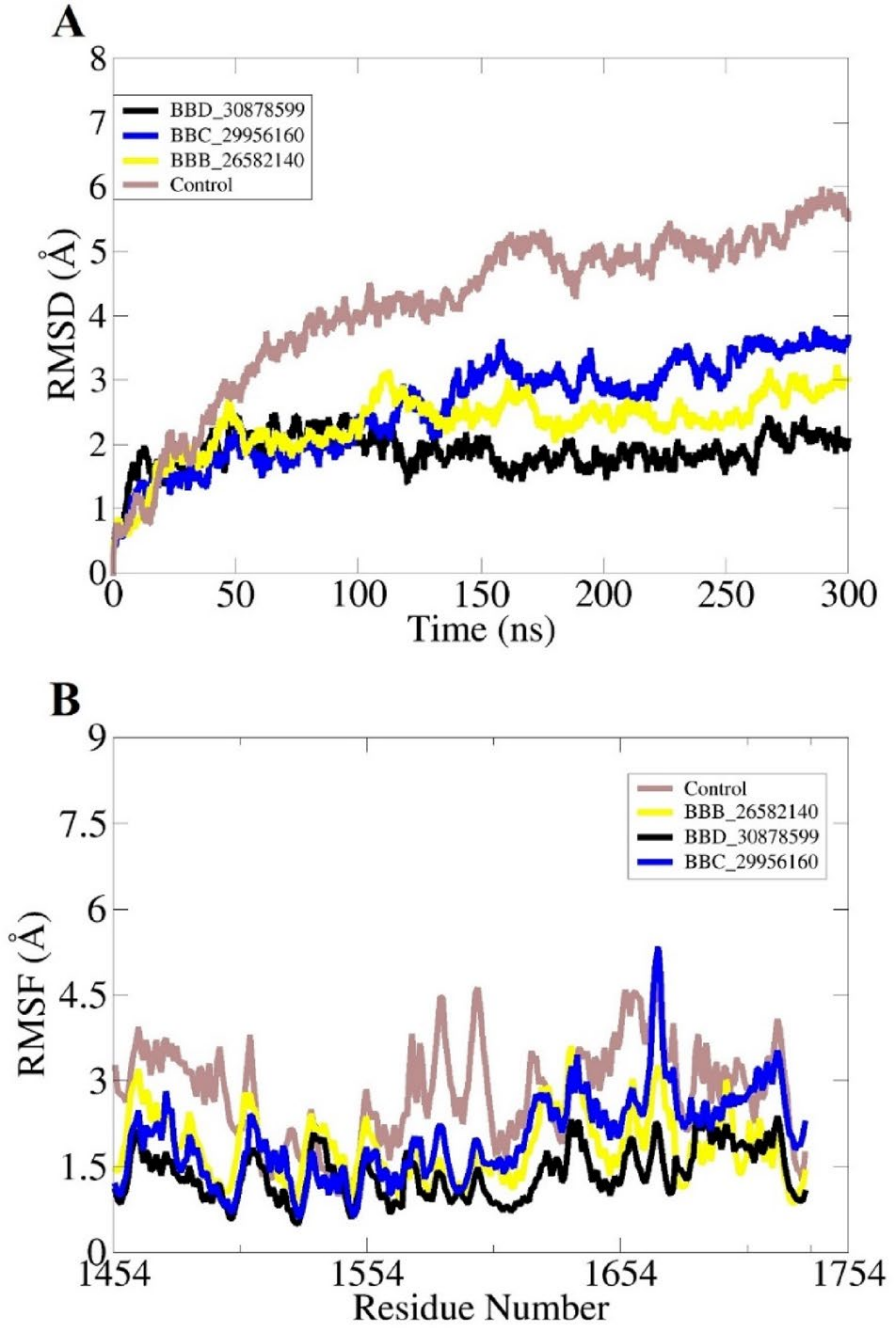
Dynamic investigation of docked complexes. (**A**) structure stability RMSD and (**B**) residue flexibility RMSF.

**Figure 6:**
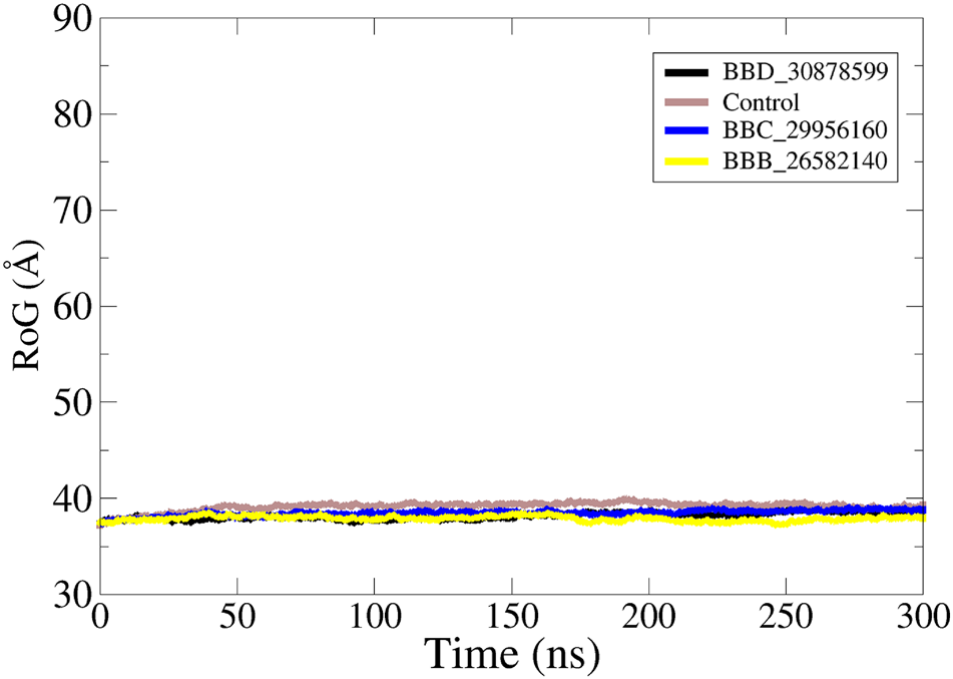
RoG analysis of docked complexes. The analysis is done considering carbon alpha atoms.

Insights about physical movements of docked compound with receptor were accomplished using molecular dynamic simulation assay. This was essential as docking studies only provide one snapshot view while biomolecules behave in time dependent dynamics milieu. The first analysis performed in this regard was root mean square deviation (RMSD), followed by root mean square fluctuation (RMSF). Both these analyses were conducted considering carbon alpha atoms of the complexes. Compared to control, all the complexes disclosed stable dynamic conformation of polyketide synthase Pks13. The RMSD of all complexes reported stable behavior in the presence of docked compounds. The most stable system is of BBD_30878599 with mean RMSD of 1.07 Å. The control system shown higher RMSD with regular variations throughout the simulation length. The mean RMSD noted for control complex was greater than 3 Å. Similarly, the RMSF complements the RMSD analysis. The N-terminal of the receptor bimolecular showed higher RMSF than the C-terminal. The RMSD and RMSF plots are given in Fig. 5. Previously, it was reported that Coumestan derivatives were able to bind well with Pks13 and induce colony-forming unit (CFU) reduction of 1.0/ml of the bacterial culture^65^. Further, it was demonstrated that Benzofuran derivatives inhibit the Pks13 enzyme both in vitro and in vivo^37^. The radius of gyration (RoG) analysis was further performed to examine the compact behavior of docked complexes^66,67^. The receptors compact behavior is important to maintain strong intermolecular docked conformation and interactions. The RoG of studied systems is given in Fig. 6. All the three systems revealed RoG value ~ 37–38 Å, revealing highly stable RoG. Similarly, solvent accessible surface area (SASA) analysis was conducted for the systems^68,69^. The SASA for BBB_26582140, BBD_30878599, BBC_29956160 and control is given S-Fig. 1, S-Fig. 2, S-Fig. 3 and S-Fig. 4, respectively. The analysis demonstrated that the intermolecular interactions is highly intact and the water molecules accessibility is low.

### MDS free energy analysis

The MM-GBSA and MM-PBSA binding free energy are undoubtedly promising methods in predicting binding affinity of compounds docked to a particular biomolecule. These methods are appreciated in term of computational speed demands and reliability in predicting compounds interaction strength compared to experimental data. In comparison to control, it has been revealed that molecule BBD_30878599 is the most promising binder to Pks13-TE with net energy value of − 79.75 kcal/mol in MM-GBSA and − 79.31 kcal/mol in MM-PBSA. Similarly, the BBB_26582140 and BBC_29956160 have also demonstrated better binding to Pks13-TE in reference to the control. The gas phase energy was found dominating, whereas van der Waals played a major role in compounds binding with Pks13-TE. This was followed by electrostatic energy. The negative contribution was seen polar solvation energy. The data reported by both MM-GBSA and MM-PBSA techniques noticed the compounds showed considerable compounds binding to the receptor biological molecule. Details about each parameter energy contribution to the net MM-GBSA and MM-PBSA are presented in Table 1. Furthermore, the binding energy score of hotspot residues involved in binding with the compounds is given in Table 2. These hotspot residues have vital contribution in overall intermolecular conformational and interaction stability^66,70^.

**Table 1:** MDS free energies analysis (kcal/mol).

Energy parameter	BBB_26582140	BBD_30878599	BBC_29956160	Control
MM-GBSA				
Van der Waals	− 66.68	− -62.08	− 61.87	− 55.68
Electrostatic	− 20.23	− 18.33	− 15.46	− 12.06
Polar	19.10	16.66	12.85	11.30
Non-polar	− 10.69	− 16.00	− 11.22	− 9.14
Gas phase	− 86.91	− 80.41	− 77.33	− 67.74
Solvation	8.41	0.66	1.63	2.16
Delta	− 78.5	− 79.75	− 75.7	-65.58
MM-PBSA				
Van der Waals	− 66.68	− 62.08	− 61.87	− 55.68
Electrostatic	− 20.23	− 18.33	− 15.46	− 12.06
Polar	20.10	15.08	13.93	12.90
Non-polar	− 10.56	− 13.98	− 10.55	− 11.77
Gas phase	− 86.91	− 80.41	− 77.33	− 67.74
Solvation	9.54	1.1	3.38	1.13
Delta	− 77.37	− 79.31	− 73.95	− 66.61

**Table 2:** Binding energy score of hotspot residues involved in compounds binding.

Residue	BBB_26582140	BBD_30878599	BBC_29956160
Ala1477	− 1.52	− 1.08	− 0.51
Arg1578	− 1.05	− 0.68	− 1.32
Tyr1582	− 8.64	− 1.34	− 0.96
Tyr1637	− 0.53	− 0.86	− 1.24
Asn1640	− 1.86	− 4.50	− 4.25
Arg1641	0.20	− 0.36	− 1.07
Asp1644	− 4.36	− 3.85	− 3.88
Ala1667	− 2.87	− 3.99	− 1.30
His1699	− 3.59	− 1.55	− 1.11
Phe1670	− 1.67	− 1.29	− 1.01

### WaterSwap binding energy

**Figure 7:**
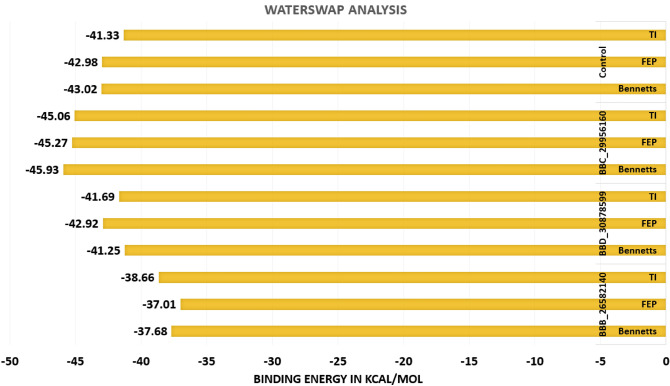
WaterSwap binding free energy in kcal/mol. The energy values are the outcome of three algorithms.

Additional confidence was accomplished on the MM-GBSA and MM-PBSA results using WaterSwap method. This method is regarded more accurate due to its ability to consider the role of water molecules particularly those that bridge the ligand with the protein residues. The WaterSwap based energy calculation was done using three algorithms; TI, FEP and Bennett’s. The difference of value 1 kcal/mol among the algorithms demonstrated the systems well converged. As can be noticed, the systems are significantly stable in term of intermolecular binding interactions and binding conformations. The WaterSwap based binding free energies are given in Fig. 7.

### Entropy energy calculation

**Figure 8:**
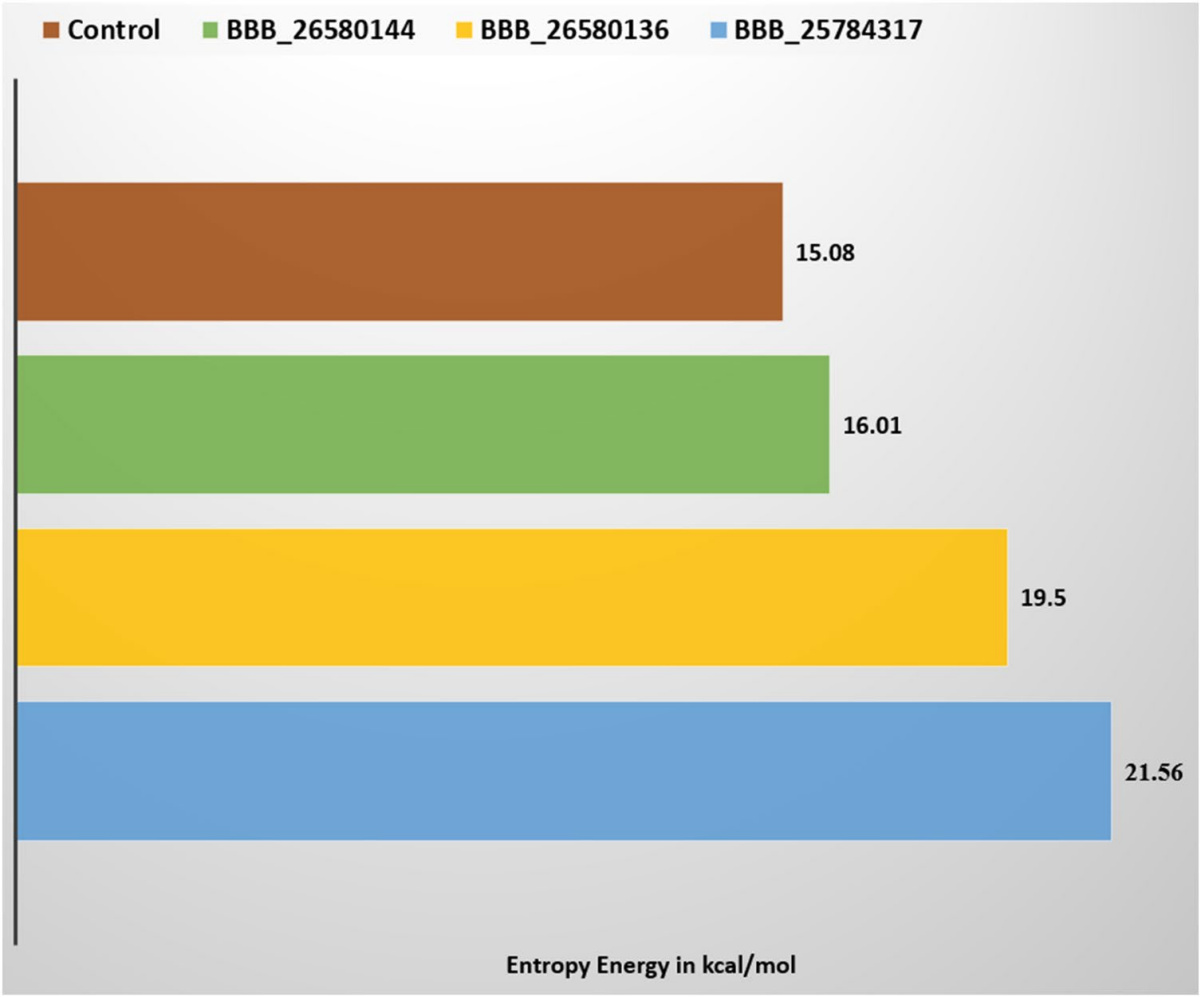
Entropy energy analysis of docked systems. All values are in kcal/mol.

Binding entropy estimation was performed to determine freedom energy of docked molecules with Pks13-TE. It was demonstrated that net entropy energy is negative in all systems, indicating that the systems significantly hold non-favorable energy that contribution to the instability of the systems. However, this energy is much less than the net total of MM-GBSA and MM-PBSA which indicates that systems are stable enough to ensure intermolecular docked conformation. The systems entropy energy is provided in Fig. 8.

### Pharmacokinetic analysis

In silico pharmacokinetic analysis is vital to shed light on what the body will do with the selected drugs. The study can guide how the drugs will be subjected to absorption, bioavailability, distribution, metabolism, and excretion once administered into the host body. In pharmacokinetic, the most significant factor to evaluate is the oral bioavailability and adsorption of the drugs in gastrointestinal tract. Except BBD_30878599, the selected compounds were predicted to show high gastrointestinal absorption including the control molecule. This indicates that high proportions of drugs can be available for therapeutic affect at the target sites. Determination of compounds permeability to the central nervous system (CNS) is prerequisite in drug discovery. All the compounds as well as the control were found non-permeable to the CNS. The P-glycoprotein 1 (Pgp-1) is a multi-drug resistance protein that pumps out foreign substances from the cells. It was found that the BBD_30878599 and control are non-substrate to Pgp-1 and thus they are unlikely to be excluded from the cells. Similarly, all the compounds are non-inhibitors of CYP1A2, CYP2C19, CYP2C9, CYP2D6 and CYP3A4. These cytochrome proteins are involved in metabolism of drugs and xenobiotic. The control is inhibitor for majority of these cytochromes. The compounds skin permeation value ranges from − 7.61 to − 8.17 cm/s compared to control (− 7.19 cm/s). The pharmacokinetic analysis of compounds is presented in Table 3. From water solubility perspective, the compounds were classified as good water soluble. The control molecule was predicted as moderate water soluble.

**Table 3:** Pharmacokinetics analysis of compounds and control.

Pharmacokinetics	BBB_26582140	BBD_30878599	BBC_29956160	Control
GI absorption	High	Low	High	High
BBB permeant	No	No	No	No
P-gp substrate	No	Yes	No	Yes
CYP1A2 inhibitor	No	No	No	Yes
CYP2C19 inhibitor	No	No	No	No
CYP2C9 inhibitor	No	No	No	Yes
CYP2D6 inhibitor	No	No	No	Yes
CYP3A4 inhibitor	No	No	No	No
Log Kp (skin permeation)	− 7.79 cm/s	− 8.17 cm/s	− 7.61 cm/s	− 7.19 cm/s

### Druglikeness/medicinal chemistry analysis

Druglike compounds have higher chances of reaching market and can be branded into a successful drug. There are certain rules that are available to check whether a compound is drug like or not. The most important is Lipinski rule of five. According to this rule, all the compound fulfills Lipinski rule of five and are classified as drug like. This rule defines a drug molecule to have molecular weight < 500 Dalton, hydrogen bond donors < 5, hydrogen bond acceptors < 10, and topological polar surface area < 140 Å^2^ and LogP value < 5. Likewise, except control molecule, Ghose predicted all the screened molecules to be non-drug as they have disagreed on the LogP and molecular weight. Similarly, the compounds agree on Veber, Egan and Muegge rules. Also, the compounds have good bioavailability score. The compounds except control was found to have zero alert for pan-assay interference (PAINS) compounds and likely to interact with one specific biomolecule. The BBB_26582140 was the only compound that has lead-like structure and thus can be structurally optimized to enhance the biological potency. The synthetic accessibility predicted the compounds can be easily synthesized for experimental analysis. All the druglikeness/medicinal chemistry analysis are presented in Table 4**.** The compounds are also classified as non-mutagenic, non-carcinogenic and non-AMES toxic. Further, the compounds showed less hepatotoxicity.

**Table 4:** Druglikeness/medicinal chemistry analysis of compounds and control.

Druglikeness/Medicinal chemistry	BBB_26582140	BBD_30878599	BBC_29956160	Control
Lipinski	Yes; 0 violation Molecular Weight (265.31 g/mol), Number of H-bond acceptors (3), Num. H-bond donors (3), TPSA (85.89 Å^2^), LogP (0.46)	Yes; 0 violation Molecular Weight (240.30 g/mol), Number of H-bond acceptors (4), Num. H-bond donors (5), TPSA (85.42 Å^2^), LogP (-0.49)	Yes; 0 violation Molecular Weight (158.16 g/mol), Number of H-bond acceptors (3), Num. H-bond donors (4), TPSA (99.84 Å^2^), LogP (-1.56)	Yes; 0 violation Molecular Weight (408.45 g/mol), Number of H-bond acceptors (6), Num. H-bond donors (3), TPSA (95.17 Å^2^), LogP (2.43)
Ghose	No; 1 violation: WLOGP < -0.4	No; 1 violation: WLOGP < -0.4	No; 3 violations: MW < 160, WLOGP < -0.4, MR < 40	Yes
Veber	Yes	Yes	Yes	Yes
Egan	Yes	Yes	Yes	Yes
Muegge	Yes	Yes	Yes	Yes
Bioavailability Score	0.55	0.55	0.55	0.55
PAINS	0 alert	0 alert	0 alert	1 alert: mannich_A
Brenk	0 alert	0 alert	0 alert	0 alert
Lead-likeness	Yes	No; 1 violation: MW < 250	No; 1 violation: MW < 250	No; 1 violation: MW > 350
Synthetic accessibility	3.02	4.15	2.54	4.10
AMES Toxicity	No	No	No	No

## Conclusions

In this study, three compounds; BBB_26582140, BBD_30878599 and BBC_29956160 were identified as best binders of Pks13-TE. The compounds were noticed to docked deep inside the binding pocket of the enzyme and formed rich pattern of both hydrophobic and hydrophilic contacts. The compounds binding conformations with the enzyme were found highly stable. This was validated by the different binding free energy methods used in the study. Also, the compounds were found to have promising druglike and pharmacokinetic properties. In a nutshell, the compounds identified herein were promising in term of affinity for the Pks13-TE and thus can be subjected to experimental evaluations. Further, the compounds might provide starting leads to speed up drug discovery and development against thioesterase domain of Polyketide synthase 13 of *M. tuberculosis*.

## Supplementary Information

Supplementary Information.

## Data Availability

All data generated or analysed during this study are included in this published article.
