# Supplementary material for: An integrated computational approach towards novel drugs discovery against polyketide synthase 13 thioesterase domain of Mycobacterium tuberculosis
Source: Sci Rep. 2023 Apr 28;13:7014. doi: 10.1038/s41598-023-34222-8 (PMC10147368; doi:10.1038/s41598-023-34222-8)
Supplement: Supplementary file 1 — Supplementary Information. [file 41598_2023_34222_MOESM1_ESM.docx]

**An Integrated Computational Approach Towards Novel Drugs Discovery Against Polyketide Synthase 13 Thioesterase domain of *Mycobacterium tuberculosis***

Ali Altharawi^1^, Manal A. Alossaimi^1^, Mohammed M. Alanazi^2^, Safar M. Alqahatani^1^ and Muhammad Tahir ul Qamar^3,*^

^1^ Department of Pharmaceutical Chemistry, College of Pharmacy, Prince Sattam Bin Abdulaziz University, Al-Kharj 11942, Saudi Arabia

^2^ Department of Pharmaceutical Chemistry, College of Pharmacy, King Saud University, Riyadh 11451, Saudi Arabia

^3^ Department of Bioinformatics and Biotechnology, Government College University Faisalabad (GCUF), Faisalabad-38000, Pakistan

***Corresponding author**: [tahirulqamar@gcuf.edu.pk](mailto:tahirulqamar@gcuf.edu.pk)


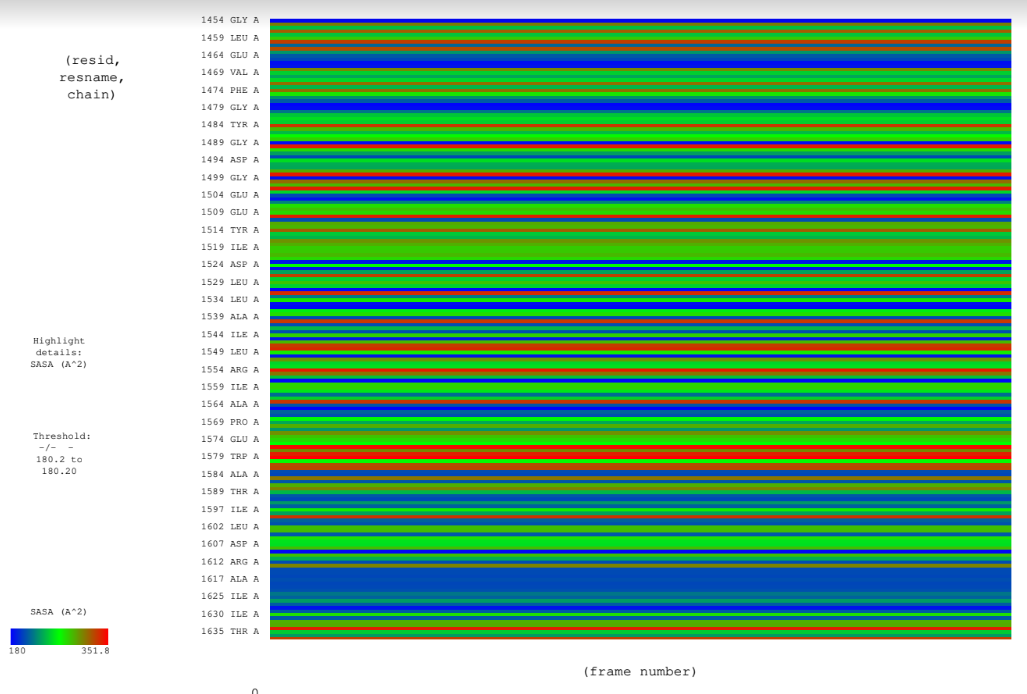


**S-Fig.1.** SASA analysis of BBB_26582140 polyketide synthase 13 thioesterase domain complex. The figure is generated by VMD 1.93 software (https://www.ks.uiuc.edu/Research/vmd/vmd-1.9.3/).


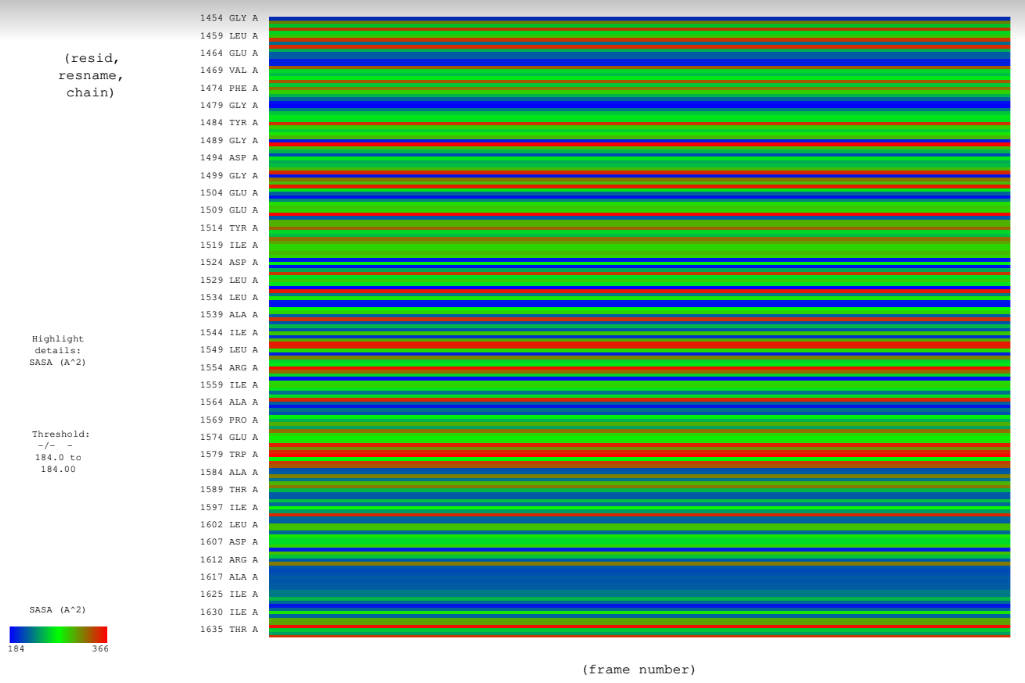


**S-Fig.2.** SASA analysis of BBD_30878599 polyketide synthase 13 thioesterase domain complex. The figure is generated by VMD 1.93 software (https://www.ks.uiuc.edu/Research/vmd/vmd-1.9.3/).


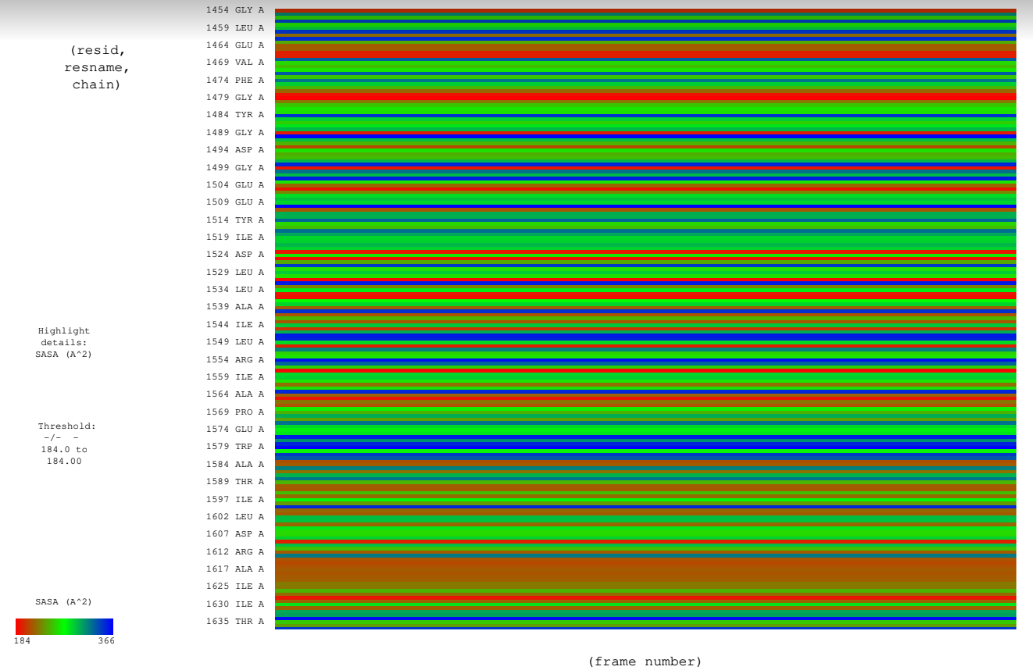


**S-Fig.3.** SASA analysis of BBC_29956160 polyketide synthase 13 thioesterase domain complex. The figure is generated by VMD 1.93 software (https://www.ks.uiuc.edu/Research/vmd/vmd-1.9.3/).


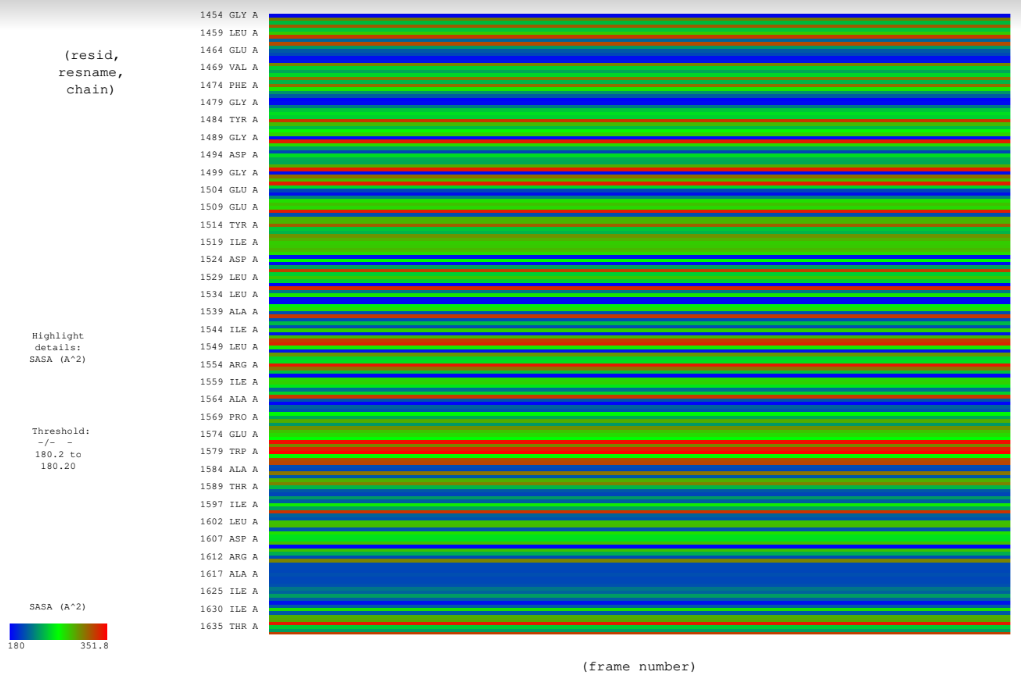


**S-Fig.4.** SASA analysis of control polyketide synthase 13 thioesterase domain complex. The figure is generated by VMD 1.93 software (https://www.ks.uiuc.edu/Research/vmd/vmd-1.9.3/).
